# Bilateral metachronous breast malignancies: Malignant phylloides and invasive breast carcinoma—a case report

**DOI:** 10.3389/fonc.2023.1034556

**Published:** 2023-03-24

**Authors:** Norlia Abdullah, Iqbal Hussain Rizuana, Janice Hui Ling Goh, Qi Zheng Lee, Nurismah Md Isa, Suria Hayati Md Pauzi

**Affiliations:** ^1^Surgery Department, Universiti Kebangsaan Malaysia Medical Centre, Cheras, Malaysia; ^2^Radiology Department, Universiti Kebangsaan Malaysia Medical Centre, Cheras, Malaysia; ^3^Pathology Department, Universiti Kebangsaan Malaysia Medical Centre, Cheras, Malaysia

**Keywords:** metachronous, malignant phylloides, breast carcinoma, mastectomy, recurrence

## Abstract

A 57-year-old Malay nullipara initially presented with a right breast lump that was increasing in size but defaulted follow-up. Two years later, she developed a contralateral breast lump. She only returned to the hospital when the right breast lump had become painful, 4 years from its onset. The biopsy of the right breast lump was a phylloides tumor and that of the left breast lump was a carcinoma. She had bilateral palpable axillary lymph nodes. She underwent bilateral mastectomy and axillary dissection. The pathology report confirmed the right breast lesion to be a malignant phylloides and the left breast lesion to be a carcinoma (pT3N2). She declined adjuvant treatment. A year after the surgical operation of the metachronous lesions, she had a right chest wall recurrence with widespread pulmonary metastases. She was given palliative chemotherapy but succumbed several months later.

## Introduction

The occurrence of bilateral breast cancer is uncommon; the incidence is 3% of all breast cancers. The second cancer may be synchronous or metachronous. Synchronous breast cancer is defined as breast cancer occurring within 1 year of the earlier cancer. Metachronous cancers develop more than a year from the initial cancer (1). There have been rare reports of synchronous phylloides tumors (PTs) with contralateral invasive carcinoma (2) and metachronous bilateral breast cancers of different histopathology (3–5).

## Case report

Madam R was a Malay nullipara, a banker, with no known medical illness. She had menarche and menopause at the age of 12 and 55 years, respectively. She had no history of oral contraceptive pill usage or hormonal replacement therapy. There was no family history of breast or ovarian malignancy.

At 57 years old, she complained of a right breast lump. A mammogram was done at the National Cancer Society Center. She was informed that there were mammogram abnormalities in the right breast. However, she defaulted follow-up and sought alternative treatment instead.

Two years later, she presented to another hospital. The right breast lump had increased in size and she was found to have a contralateral breast lump. A mammogram with complementary breast and axillary ultrasound showed bilateral BI-RADS 5 lesions. A core-needle biopsy showed the right breast lump to be a PT, but the left breast lump was reported to be benign. As the left breast biopsy may have missed the lesion because it was discordant with the imaging findings, she was advised to undergo a repeat biopsy but she declined. Again, she defaulted follow-up.

At 61 years old, the patient came to our hospital due to pain in the right breast lump. On clinical examination, there was a hard bosselated mass occupying the whole right breast measuring 12 cm × 13 m with a right axillary lymph node measuring 1.5 cm. On the contralateral breast, there was an upper inner quadrant mass measuring 4.5 cm × 6.8 cm with a left axillary lymph node measuring 1 cm.

A mammogram of the left breast showed a lobulated mass with clustered microcalcifications at the upper inner quadrant with associated architectural distortion. Core-needle biopsy was repeated for the left breast lump and reported to be a left invasive carcinoma, no special type, ER positive >95%, PR positive 30%, and HER2 negative 1+.

**Figure 1:**
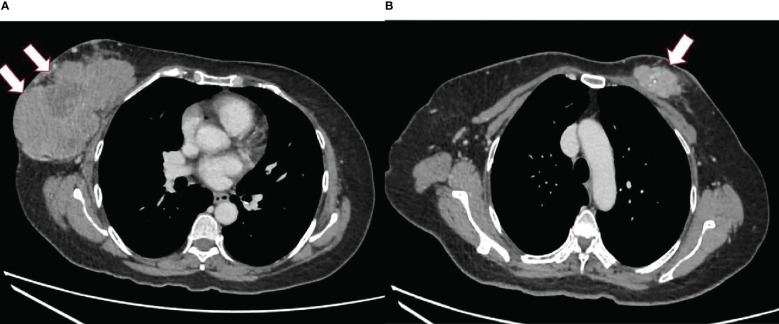
CT scan of the thorax. **(A)** Right breast mass, arrowed. **(B)** Left breast mass, arrowed.

Computed tomography (CT) of the thorax, abdomen, and pelvis was done to stage the disease. The CT scan revealed a well-defined lobulated heterogeneously enhancing mass at the retroareolar and outer half of the right breast measuring 7.9 × 13.3 × 10.5 cm with a necrotic center and foci of calcifications within. The right nipple was retracted and the skin was thickened. The mass was abutting the right pectoralis muscles with no clear fat plane at the central region (**Figure 1A**). A lobulated heterogeneously enhancing mass at the upper inner quadrant of the left breast extended to the retroareolar region, measuring 2.4 × 5.4 × 4.2 cm with calcifications and a necrotic center within (**Figure 1B**). There were bilateral enlarged axillary lymph nodes.

**Figure 2:**
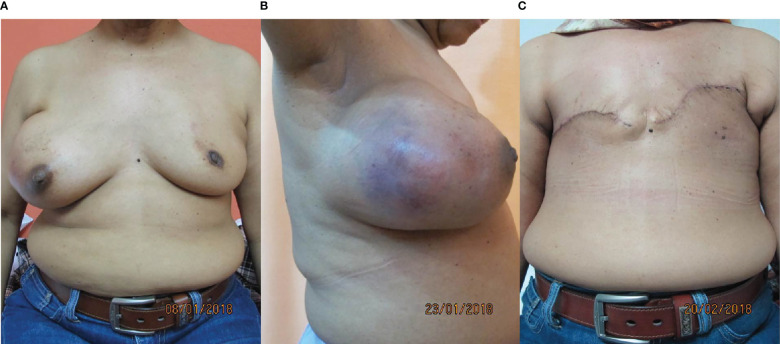
Photos of the patient. **(A, B)** Preoperatively, front and right lateral views. **(C)** Postoperatively, front.

**Figure 3:**
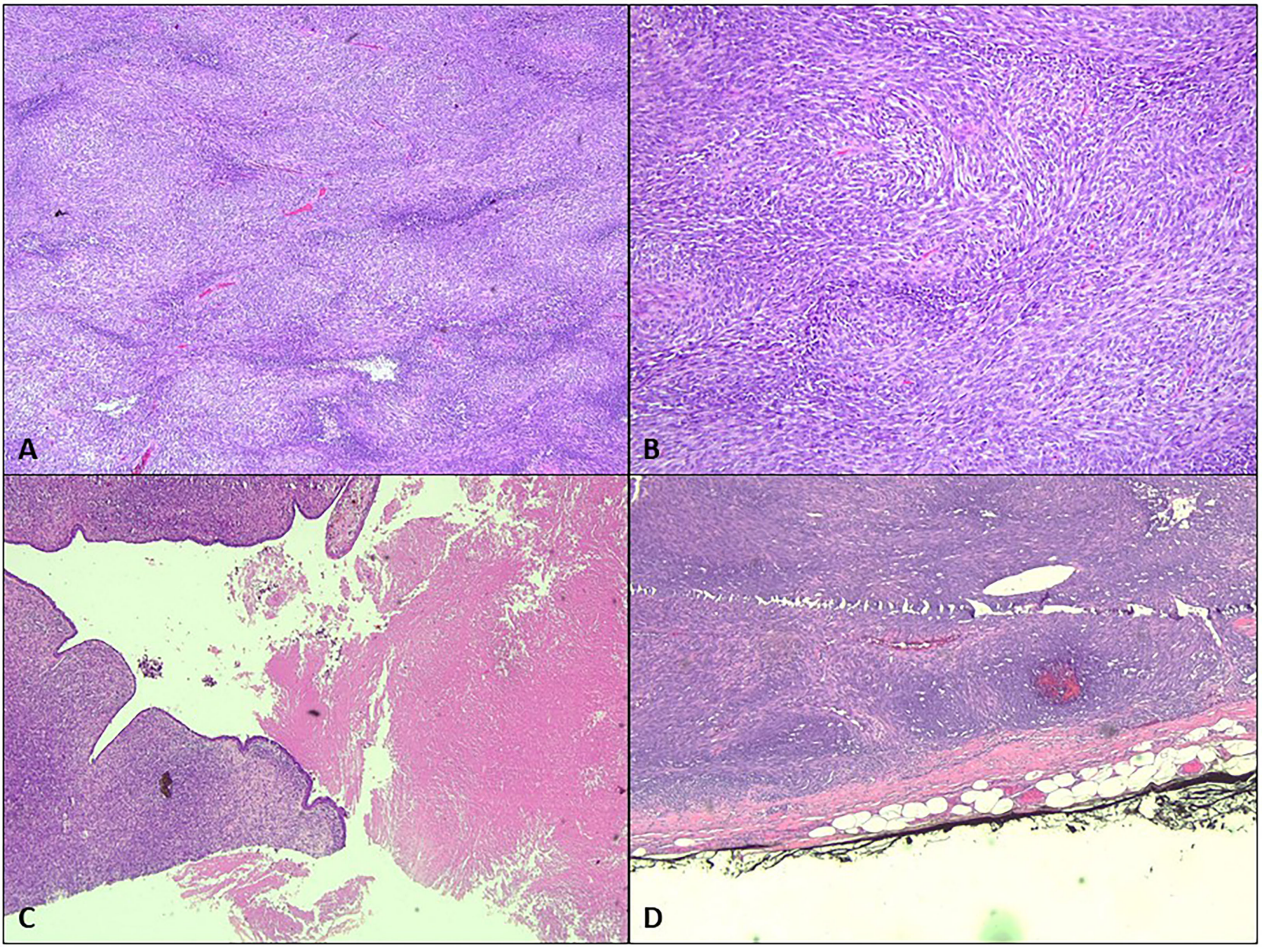
Microscopic analysis of the right breast. **(A)** The right breast tumor shows hypercellularity [hematoxylin–eosin (H&E) staining, ×4]. **(B)** The spindle-shaped cells are arranged in fascicles in a herringbone pattern (H&E, ×20). **(C)** Focal leaf-like pattern typical of phylloides tumor with adjacent necrosis (H&E, ×4). **(D)** The tumor is seen less than 1 mm from the inked deep margin (H&E, ×4).

**Figure 4:**
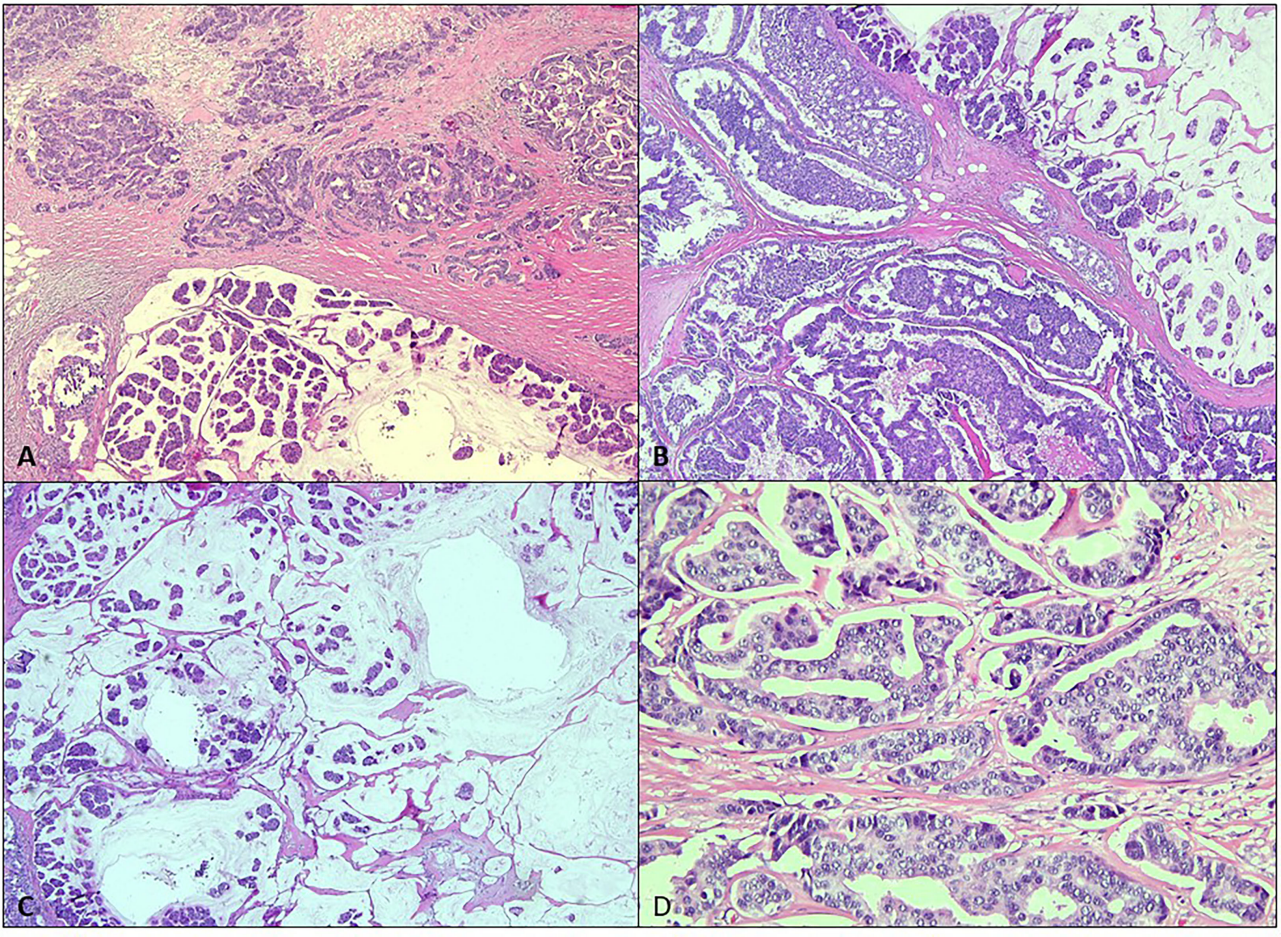
Microscopic analysis of the left breast. **(A, B)** The left breast tumor shows variable morphology of invasive carcinoma comprised of infiltrating small ducts, cords, and cribriform glands with a micropapillary pattern (H&E, ×4). **(C)** Clusters of cells floating in mucin pools (H&E, ×4). **(D)** The malignant cells display pleomorphic vesicular nuclei with inconspicuous nucleoli (H&E, ×20).

The patient underwent bilateral mastectomy and axillary dissection successfully (**Figures 2A-C**). Intraoperatively, the masses were not attached to the underlying pectoralis muscle and were easily removed from the pectoralis fascia. The right mastectomy specimen weighed 1.2 kg with a maximal diameter of 16 cm and was a malignant phylloides (**Figure 3**). A right axillary dissection was performed as the lymph nodes were palpable, but all 18 lymph nodes were clear of malignancy. The left mastectomy specimen weighed 400 g. The lump had a diameter of 6 cm. It was an invasive carcinoma of no special type (ER positive 60%, PR positive 50%, and HER2 negative) (**Figure 4**). The left axillary dissection revealed 7 positive out of a total of 13 lymph nodes. Microscopically, the closest surgical margin for each tumor was the deep margin measuring 1 mm.

Unfortunately, the patient declined all adjuvant therapy despite repeated advice. A year after the operation, she developed an enlarging right chest wall lump. When she came, it measured 10 cm which was biopsied and confirmed to be a recurrent malignant PT. There were also right palpable axillary lymph nodes. A CT scan of the thorax demonstrated a large right chest wall mass, right axillary lymph nodes measuring 3.1 × 4.9 × 6.8 cm, and multiple lung nodules of varying sizes.

She underwent several cycles of palliative chemotherapy (paclitaxel) but had neutropenic sepsis with obvious disease progression. She declined further chemotherapy and was just given oral endocrine treatment (letrozole) for several weeks. She succumbed soon after due to widespread pulmonary metastases.

## Discussion

PTs, originally known as cystosarcoma phylloides, are rare fibroepithelial breast tumors. They are characterized by the proliferation of epithelial and stromal components. They constitute less than 1% of breast neoplasms (6) and are more common in the fifth decade of life, as in this patient. They are characterized by rapidly growing breast lumps with a median symptom duration of 2 months (7). The WHO has classified PTs into benign, borderline (low-grade malignant), and malignant (high-grade malignant) based on histology, and they constitute 52.3%–74.6%, 11.1%–16.1%, and 9.3%–20% of cases, respectively (6).

PTs appear as well-circumscribed round or oval lobulated masses on mammogram. They occasionally contain calcifications. While on ultrasound, they appear as well-circumscribed round or oval lobulated hypoechoic, solid masses. Scattered cystic components may be seen at times. There are no distinct imaging characteristics to reliably distinguish benign from malignant PTs. However, features that may raise a suspicion of malignancy include branching, segmental or pleomorphic-type microcalcifications and spiculated masses on mammogram, and increased height–width ratio of irregular or ill-defined lesions on ultrasound (8).

In our patient, her initial right breast biopsy showed epithelial and connective tissue hyperplasia with no evidence of malignancy, with features favoring a benign PT. However, 2 years later, the right breast (mastectomy specimen) had transformed into a malignant PT with fibrosarcomatous elements.

Malignant transformation usually arises from the stromal components (6). For this patient, the right breast tumor showed malignant features such are hypercellularity, composed of spindle-shaped cells arranged in fascicles in a herringbone and a focal leaf-like pattern typical of PT with adjacent necrosis. The left breast tumor showed the morphology of invasive carcinoma comprised of infiltrating small ducts, cords, and cribriform glands with a micropapillary pattern and clusters of cells floating in mucin pools.

There were several reports of synchronous PTs with contralateral invasive carcinoma but only one case was reported to be metachronous (2, 7). We believe this is only the second of such a case reported in the literature.

Treatment of PTs is generally wide local excision, or mastectomy, with a minimum margin of 1 cm. Adequate clear margins can prevent a recurrence, especially in borderline and malignant PTs (8). PTs typically behave more like sarcomas and do not metastasize to regional lymph nodes (9). In this case, for PT in the right breast, mastectomy and axillary lymph node dissection were performed, due to the size of the tumor which occupied the whole breast and the palpable ipsilateral right axillary lymph nodes. Mastectomy and axillary dissection were performed on the contralateral breast (invasive carcinoma) with palpable lymph nodes. She would have benefitted from prompt adjuvant chemotherapy, radiotherapy, and endocrine therapy to treat the left breast carcinoma with high nodal involvement. Adjuvant chemotherapy is usually reserved for metastatic malignant phylloides. Radiotherapy has a role in the treatment of malignant phylloides and carcinoma when there are involved margins, or margins less than 1 cm for malignant phylloides (10). As the two malignancies respond differently to the various treatment modalities, each lesion has to be dealt with individually, as stated above.

Postoperatively, the risk of local recurrence for PTs is reported as 8% in 10 years with adequate excised margin (2). Unfortunately, for this patient, both tumors had close surgical margins. This made the local recurrence risk of the PT to be high as the surgical margin was less than 1 cm. Hence, regular clinical and annual mammograms, in cases of breast-conserving surgery, are essential to detect early signs of local recurrence.

It is reported that distant metastases (brain and lungs) occur in 10% of malignant PTs. Once metastases have occurred, they indicate a poor prognosis as they respond to systemic therapy poorly (8). The survival outcomes for those with metastatic disease from a breast carcinoma are better than those from a malignant PT. There has been no single publication comparing survival data head-to-head between breast carcinoma and malignant phylloides. For those with stage 4 breast carcinoma, the 5-year survival has been found to vary from 15% to 27% (11). However, the median overall survival of those with metastatic malignant PT has been reported to be only 10.7 months (12).

The coexistence of invasive breast carcinoma and PTs in the ipsilateral breast has been described in the literature; it is hypothesized that this could be due to the invasive breast carcinoma cells arising from the adjacent tissue or within the phylloides tissues. Mathias et al. (2) postulated that the coexistence of contralateral invasive breast carcinoma and PTs could be associated with germline mutations (*PTEN* and *PARP4* genes) and exposure to radiotherapy. Both of these factors increase the risks of metachronous and synchronous malignancies. However, our patient was not known to have any germline mutation and was neither exposed to prior radiotherapy.

Similar to this patient, there is a small but significant percentage of the Malay population in Malaysia who tend to favor alternative treatment and present themselves late to the hospital which results in a poor clinical outcome (13) despite ongoing public education efforts by the government and non-governmental agencies.

## Conclusion

Metachronous and synchronous contralateral malignant phylloides and invasive breast carcinoma are rare. However, clinicians and pathologists need to be aware of the possibility of two different pathologies existing when dealing with bilateral breast disease, as in this case, as the required treatment options and eventual outcomes differ. More studies need to be done in order to understand the histopathological events better.

## Data availability statement

The original contributions presented in the study are included in the article/supplementary material. Further inquiries can be directed to the corresponding author.

## Ethics statement

Written consent was obtained from the patient for publication of this case report.

## Author contributions

NA was responsible for the final editing, provided the patient’s photos, and acted as the main clinician. IHR reported the images and edited the draft. JG wrote the initial draft. QL co-wrote the initial draft. NMI and SMP prepared and reported the pathology slides. All authors contributed to the article and approved the submitted version.
